# 4′-(4-Bromo­phen­yl)-1′-methyl­dispiro­[indan-2,2′-pyrrolidine-3′,2′′-indan]-1,3,1′′-trione

**DOI:** 10.1107/S1600536811044515

**Published:** 2011-10-29

**Authors:** Ang Chee Wei, Mohamed Ashraf Ali, Rusli Ismail, Madhukar Hemamalini, Hoong-Kun Fun

**Affiliations:** a Institute for Research in Molecular Medicine, Universiti Sains Malaysia, 11800 USM, Penang, Malaysia; b X-ray Crystallography Unit, School of Physics, Universiti Sains Malaysia, 11800 USM, Penang, Malaysia

## Abstract

In the title compound, C_27_H_20_BrNO_3_, the pyrrolidine ring adopts a half-chair conformation, while the other five-membered rings adopt flattened envelope conformations with the spiro C atoms as the flap atoms. An intra­molecular C—H⋯O hydrogen bond occurs, generating an *S*(6) ring. In the crystal, mol­ecules are connected *via* weak C—H⋯O hydrogen bonds, forming chains along the *c* axis.

## Related literature

For background to tuberculosis, see: Sunduru *et al.* (2010 ▶); Trivedi *et al.* (2010 ▶). For background to anti-tuberculous drugs, see: Moraski *et al.* (2011 ▶); Kumar *et al.* (2009 ▶); Maheswari *et al.* (2010 ▶). For puckering parameters, see: Cremer & Pople (1975 ▶). For the stability of the temperature controller used in the data collection, see: Cosier & Glazer (1986 ▶).
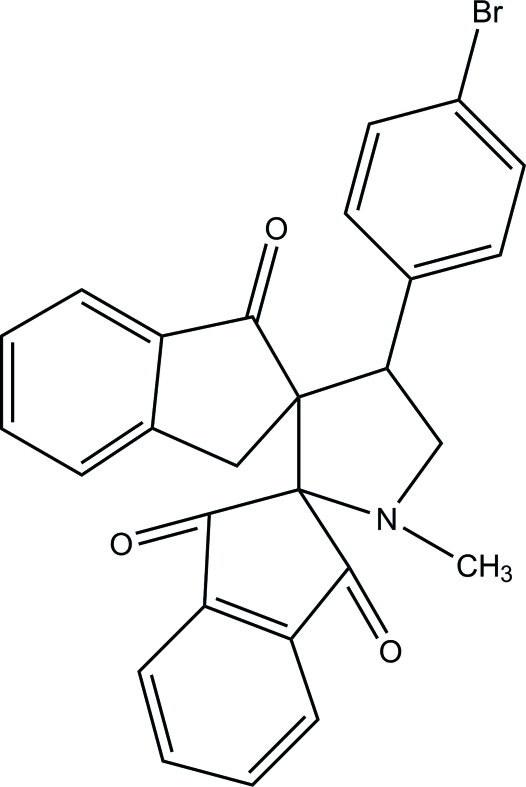


## Experimental

### 

#### Crystal data

C_27_H_20_BrNO_3_
*M* *_r_* = 486.35
Monoclinic, 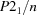

*a* = 7.8392 (1) Å
*b* = 21.5849 (5) Å
*c* = 12.7823 (3) Å
β = 98.422 (1)°
*V* = 2139.55 (8) Å^3^
*Z* = 4
Mo *K*α radiation
μ = 1.95 mm^−1^
*T* = 100 K
0.42 × 0.20 × 0.13 mm

#### Data collection

Bruker SMART APEXII CCD diffractometer
Absorption correction: multi-scan (*SADABS*; Bruker, 2009 ▶) *T*_min_ = 0.492, *T*_max_ = 0.788
18795 measured reflections
6300 independent reflections
4709 reflections with *I* > 2σ(*I*)
*R*_int_ = 0.036

#### Refinement

*R*[*F* ^2^ > 2σ(*F* ^2^)] = 0.041
*wR*(*F* ^2^) = 0.094
*S* = 1.02
6300 reflections
290 parameters
H-atom parameters constrained
Δρ_max_ = 0.78 e Å^−3^
Δρ_min_ = −0.72 e Å^−3^

### 

Data collection: *APEX2* (Bruker, 2009 ▶); cell refinement: *SAINT* (Bruker, 2009 ▶); data reduction: *SAINT*; program(s) used to solve structure: *SHELXTL* (Sheldrick, 2008 ▶); program(s) used to refine structure: *SHELXTL*; molecular graphics: *SHELXTL*; software used to prepare material for publication: *SHELXTL* and *PLATON* (Spek, 2009 ▶).

## Supplementary Material

Crystal structure: contains datablock(s) global, I. DOI: 10.1107/S1600536811044515/hb6460sup1.cif

Structure factors: contains datablock(s) I. DOI: 10.1107/S1600536811044515/hb6460Isup2.hkl

Additional supplementary materials:  crystallographic information; 3D view; checkCIF report

## Figures and Tables

**Table 1:** Hydrogen-bond geometry (Å, °)

*D*—H⋯*A*	*D*—H	H⋯*A*	*D*⋯*A*	*D*—H⋯*A*
C11—H11*B*⋯O1	0.99	2.40	3.053 (2)	123
C15—H15*A*⋯O2^i^	0.95	2.44	3.163 (3)	133
C22—H22*A*⋯O3^i^	0.95	2.57	3.225 (3)	126

Symmetry code: (i) 
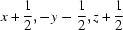
.

**Fig. 1.**
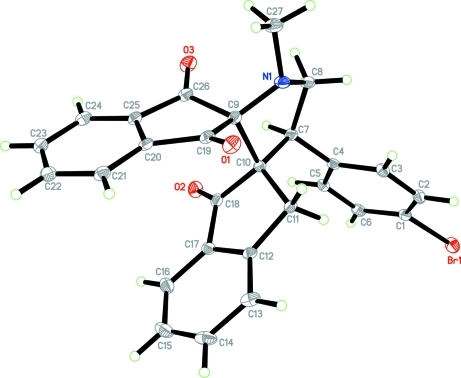
The asymmetric unit of the title compound, showing 30% probability displacement ellipsoids.

**Fig. 2.**
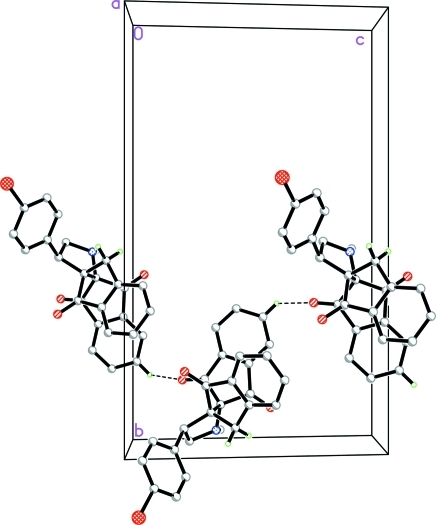
The crystal packing of the title compound (I). H atoms not involved in hydrogen bonding are omitted.

## supplementary crystallographic information

### Comment

Tuberculosis (TB) is one of the most common chronic infections caused by Mycobacterium tuberculosis and is the world's second common cause of death from infectious diseases after AIDS (Sunduru *et al.*, 2010). Among HIV infected patients, TB is the leading killer epidemic. About 2 million people living with HIV/AIDS die from TB every year (Trivedi *et al.*, 2010). To make things even worse, the emergence of multiple drug resistant TB (MDR-TB), extensively drug resistant TB (XDR-TB) and extremely drug resistant TB (XXDR-TB) are on the rise (Moraski *et al.*, 2011). These lead to the need for development of more potent drugs of new structures and with novel mechanisms of action (Trivedi *et al.*, 2010). In our study, spiro nuclei were used as the core structure due to their potential antimycobacterial properties. Some studies have shown that many of these compounds displayed comparable or even better activities than some of the first-line TB drugs (Kumar *et al.*, 2009; Maheswari *et al.*, 2010).

The asymmetric unit of the title compound is shown in Fig. 1. The pyrrolidine ring (N1/C7–C10) is twisted about the C9–C10 bond, with puckering parameters (Cremer & Pople, 1975) Q = 0.449 (2) Å and f = 317.8 (3)°, and adopting a half-chair conformation. The two five- membered carbocyclic rings, C9/C19,C20/C25,C26 and C10–C12/C17–C18, are in envelope conformations: puckering parameters Q = 0.222 (2) Å and f = 182.7 (5)°  with atom C9 at the flap; and Q = 0.216 (2) Å and f = 4.9 (5)° with atom C10 at the flap, respectively.

In the crystal packing, (Fig. 2), the molecules are connected *via* weak intermolecular C—H···O (Table 1) hydrogen bonds, forming one-dimensional chains along the *c*-axis.

### Experimental

A mixture of (*E*)-2-(4-bromobenzylidene)-2,3-dihydro-1*H*-indene-1- one (0.001 mmol), ninhydrin (0.001 mmol) and sarcosine (0.002 mmol) (1:1:2) were dissolved in methanol (10 ml) and refluxed for 4 h. After completion of the reaction as evident from TLC, the mixture was poured into crushed ice. The precipitated solid was filtered, washed with water and recrystallised from petroleum ether–ethyl acetate mixture (1:1) to reveal the title compound as yellow blocks.

### Refinement

All hydrogen atoms were positioned geometrically [ C–H = 0.95–1.00 Å] and were refined using a riding model, with *U*_iso_(H) = 1.2 *U*_eq_(C).

### Crystal data

**Table:**

C_27_H_20_BrNO_3_	*F*(000) = 992
*M**_r_* = 486.35	*D*_x_ = 1.510 Mg m^−^^3^
Monoclinic, *P*2_1_/*n*	Mo *K*α radiation, λ = 0.71073 Å
Hall symbol: -P 2yn	Cell parameters from 5478 reflections
*a* = 7.8392 (1) Å	θ = 2.5–29.8°
*b* = 21.5849 (5) Å	µ = 1.95 mm^−^^1^
*c* = 12.7823 (3) Å	*T* = 100 K
β = 98.422 (1)°	Block, yellow
*V* = 2139.55 (8) Å^3^	0.42 × 0.20 × 0.13 mm
*Z* = 4	

### Data collection

**Table:**

Bruker SMART APEXII CCD diffractometer	6300 independent reflections
Radiation source: fine-focus sealed tube	4709 reflections with *I* > 2σ(*I*)
graphite	*R*_int_ = 0.036
φ and ω scans	θ_max_ = 30.2°, θ_min_ = 1.9°
Absorption correction: multi-scan (*SADABS*; Bruker, 2009)	*h* = −11→11
*T*_min_ = 0.492, *T*_max_ = 0.788	*k* = −30→21
18795 measured reflections	*l* = −18→18

### Refinement

**Table:**

Refinement on *F*^2^	Primary atom site location: structure-invariant direct methods
Least-squares matrix: full	Secondary atom site location: difference Fourier map
*R*[*F*^2^ > 2σ(*F*^2^)] = 0.041	Hydrogen site location: inferred from neighbouring sites
*wR*(*F*^2^) = 0.094	H-atom parameters constrained
*S* = 1.02	*w* = 1/[σ^2^(*F*_o_^2^) + (0.0387*P*)^2^ + 1.0757*P*] where *P* = (*F*_o_^2^ + 2*F*_c_^2^)/3
6300 reflections	(Δ/σ)_max_ < 0.001
290 parameters	Δρ_max_ = 0.78 e Å^−^^3^
0 restraints	Δρ_min_ = −0.72 e Å^−^^3^

### Special details

**Table:**

Experimental. The crystal was placed in the cold stream of an Oxford Cryosystems Cobra open-flow nitrogen cryostat (Cosier & Glazer, 1986) operating at 100.0 (1) K.
Geometry. All s.u.'s (except the s.u. in the dihedral angle between two l.s. planes) are estimated using the full covariance matrix. The cell s.u.'s are taken into account individually in the estimation of s.u.'s in distances, angles and torsion angles; correlations between s.u.'s in cell parameters are only used when they are defined by crystal symmetry. An approximate (isotropic) treatment of cell s.u.'s is used for estimating s.u.'s involving l.s. planes.
Refinement. Refinement of F^2^ against ALL reflections. The weighted R-factor wR and goodness of fit S are based on F^2^, conventional R-factors R are based on F, with F set to zero for negative F^2^. The threshold expression of F^2^ > 2σ(F^2^) is used only for calculating R-factors(gt) etc. and is not relevant to the choice of reflections for refinement. R-factors based on F^2^ are statistically about twice as large as those based on F, and R- factors based on ALL data will be even larger.

### Fractional atomic coordinates and isotropic or equivalent isotropic displacement parameters (Å^2^)

**Table:**

	*x*	*y*	*z*	*U*_iso_*/*U*_eq_	
Br1	0.43113 (3)	0.100889 (10)	0.065729 (18)	0.03000 (8)	
O1	−0.22936 (19)	−0.10044 (7)	0.55329 (11)	0.0260 (3)	
O2	−0.0213 (2)	−0.18954 (7)	0.22339 (11)	0.0276 (3)	
O3	−0.4454 (2)	−0.15915 (7)	0.21003 (11)	0.0290 (4)	
N1	−0.3407 (2)	−0.04677 (8)	0.34244 (13)	0.0215 (4)	
C1	0.2608 (2)	0.05064 (9)	0.11634 (15)	0.0204 (4)	
C2	0.1755 (3)	0.07378 (10)	0.19495 (16)	0.0219 (4)	
H2A	0.2049	0.1132	0.2254	0.026*	
C3	0.0460 (3)	0.03840 (9)	0.22887 (15)	0.0203 (4)	
H3A	−0.0139	0.0542	0.2826	0.024*	
C4	0.0021 (2)	−0.01977 (9)	0.18553 (14)	0.0176 (4)	
C5	0.0949 (3)	−0.04220 (9)	0.10800 (15)	0.0203 (4)	
H5A	0.0685	−0.0821	0.0785	0.024*	
C6	0.2249 (3)	−0.00746 (10)	0.07308 (15)	0.0222 (4)	
H6A	0.2876	−0.0233	0.0207	0.027*	
C7	−0.1388 (2)	−0.05921 (9)	0.21992 (15)	0.0188 (4)	
H7A	−0.1739	−0.0898	0.1621	0.023*	
C8	−0.3039 (3)	−0.02450 (10)	0.23916 (16)	0.0224 (4)	
H8A	−0.2846	0.0208	0.2408	0.027*	
H8B	−0.4008	−0.0342	0.1828	0.027*	
C9	−0.2654 (2)	−0.10771 (9)	0.35915 (15)	0.0185 (4)	
C10	−0.0832 (2)	−0.09696 (9)	0.32268 (15)	0.0170 (4)	
C11	0.0410 (2)	−0.06671 (9)	0.41356 (15)	0.0182 (4)	
H11A	0.1157	−0.0356	0.3859	0.022*	
H11B	−0.0239	−0.0464	0.4650	0.022*	
C12	0.1463 (2)	−0.12043 (9)	0.46402 (15)	0.0187 (4)	
C13	0.2532 (3)	−0.12312 (11)	0.56122 (16)	0.0259 (5)	
H13A	0.2694	−0.0878	0.6058	0.031*	
C14	0.3353 (3)	−0.17868 (12)	0.59126 (17)	0.0320 (5)	
H14A	0.4071	−0.1812	0.6578	0.038*	
C15	0.3152 (3)	−0.23066 (12)	0.52644 (18)	0.0316 (5)	
H15A	0.3717	−0.2682	0.5495	0.038*	
C16	0.2136 (3)	−0.22802 (10)	0.42880 (17)	0.0259 (4)	
H16A	0.2018	−0.2629	0.3830	0.031*	
C17	0.1288 (2)	−0.17243 (9)	0.39946 (15)	0.0192 (4)	
C18	0.0067 (2)	−0.15870 (9)	0.30345 (15)	0.0193 (4)	
C19	−0.2491 (2)	−0.13185 (10)	0.47401 (15)	0.0203 (4)	
C20	−0.2659 (2)	−0.20008 (10)	0.46840 (15)	0.0193 (4)	
C21	−0.2248 (3)	−0.24379 (10)	0.54822 (16)	0.0235 (4)	
H21A	−0.1788	−0.2315	0.6180	0.028*	
C22	−0.2525 (3)	−0.30551 (11)	0.52318 (17)	0.0270 (5)	
H22A	−0.2236	−0.3362	0.5761	0.032*	
C23	−0.3230 (3)	−0.32353 (10)	0.42047 (18)	0.0274 (5)	
H23A	−0.3429	−0.3662	0.4053	0.033*	
C24	−0.3641 (3)	−0.28024 (10)	0.34061 (17)	0.0247 (4)	
H24A	−0.4117	−0.2926	0.2711	0.030*	
C25	−0.3334 (2)	−0.21820 (10)	0.36564 (15)	0.0202 (4)	
C26	−0.3611 (3)	−0.16237 (10)	0.29702 (15)	0.0214 (4)	
C27	−0.5213 (3)	−0.04025 (11)	0.35822 (17)	0.0282 (5)	
H27A	−0.5346	−0.0545	0.4294	0.042*	
H27B	−0.5946	−0.0652	0.3056	0.042*	
H27C	−0.5556	0.0034	0.3503	0.042*	

### Atomic displacement parameters (Å^2^)

**Table:**

	*U*^11^	*U*^22^	*U*^33^	*U*^12^	*U*^13^	*U*^23^
Br1	0.02737 (12)	0.02655 (12)	0.03979 (13)	0.00148 (9)	0.01733 (9)	0.00830 (10)
O1	0.0272 (8)	0.0313 (8)	0.0191 (7)	−0.0046 (7)	0.0025 (6)	−0.0031 (6)
O2	0.0340 (9)	0.0244 (8)	0.0241 (7)	−0.0011 (7)	0.0026 (6)	−0.0056 (6)
O3	0.0289 (8)	0.0354 (9)	0.0200 (7)	−0.0075 (7)	−0.0057 (6)	0.0027 (6)
N1	0.0155 (8)	0.0263 (9)	0.0232 (8)	0.0029 (7)	0.0047 (7)	0.0042 (7)
C1	0.0166 (9)	0.0225 (10)	0.0228 (10)	0.0027 (8)	0.0052 (7)	0.0073 (8)
C2	0.0239 (10)	0.0184 (10)	0.0240 (10)	0.0013 (8)	0.0056 (8)	0.0018 (8)
C3	0.0213 (10)	0.0209 (10)	0.0203 (9)	0.0041 (8)	0.0079 (8)	0.0013 (8)
C4	0.0163 (9)	0.0194 (9)	0.0164 (8)	0.0026 (7)	0.0001 (7)	0.0046 (7)
C5	0.0214 (10)	0.0198 (10)	0.0192 (9)	0.0039 (8)	0.0012 (8)	0.0005 (8)
C6	0.0223 (10)	0.0253 (10)	0.0199 (9)	0.0067 (8)	0.0061 (8)	0.0020 (8)
C7	0.0169 (9)	0.0202 (10)	0.0187 (9)	0.0002 (8)	0.0009 (7)	0.0021 (7)
C8	0.0166 (10)	0.0268 (11)	0.0236 (10)	0.0014 (8)	0.0021 (8)	0.0071 (8)
C9	0.0180 (9)	0.0207 (10)	0.0163 (9)	−0.0016 (8)	0.0009 (7)	0.0013 (7)
C10	0.0150 (9)	0.0180 (9)	0.0177 (8)	−0.0012 (7)	0.0011 (7)	0.0015 (7)
C11	0.0164 (9)	0.0186 (10)	0.0191 (9)	−0.0025 (7)	0.0010 (7)	−0.0015 (7)
C12	0.0140 (9)	0.0244 (10)	0.0178 (9)	−0.0021 (8)	0.0024 (7)	0.0024 (8)
C13	0.0187 (10)	0.0400 (13)	0.0190 (10)	−0.0028 (9)	0.0027 (8)	−0.0010 (9)
C14	0.0204 (11)	0.0530 (16)	0.0223 (11)	0.0053 (10)	0.0017 (8)	0.0125 (10)
C15	0.0246 (11)	0.0371 (13)	0.0341 (12)	0.0091 (10)	0.0077 (9)	0.0167 (10)
C16	0.0248 (11)	0.0234 (11)	0.0313 (11)	0.0036 (9)	0.0095 (9)	0.0064 (9)
C17	0.0150 (9)	0.0223 (10)	0.0209 (9)	−0.0006 (8)	0.0048 (7)	0.0044 (8)
C18	0.0182 (9)	0.0185 (9)	0.0215 (9)	−0.0037 (8)	0.0039 (8)	0.0010 (8)
C19	0.0142 (9)	0.0274 (11)	0.0191 (9)	−0.0038 (8)	0.0022 (7)	0.0016 (8)
C20	0.0128 (9)	0.0251 (10)	0.0197 (9)	−0.0036 (8)	0.0011 (7)	0.0011 (8)
C21	0.0188 (10)	0.0320 (12)	0.0189 (9)	−0.0050 (8)	0.0001 (8)	0.0042 (8)
C22	0.0212 (10)	0.0300 (12)	0.0288 (11)	−0.0023 (9)	0.0009 (9)	0.0075 (9)
C23	0.0208 (10)	0.0233 (11)	0.0374 (12)	−0.0034 (8)	0.0020 (9)	−0.0006 (9)
C24	0.0188 (10)	0.0297 (11)	0.0247 (10)	−0.0044 (9)	0.0005 (8)	−0.0030 (9)
C25	0.0161 (9)	0.0249 (10)	0.0194 (9)	−0.0039 (8)	0.0019 (7)	0.0018 (8)
C26	0.0177 (9)	0.0260 (11)	0.0198 (9)	−0.0037 (8)	0.0010 (8)	0.0005 (8)
C27	0.0194 (10)	0.0368 (13)	0.0293 (11)	0.0021 (9)	0.0066 (8)	0.0039 (10)

### Geometric parameters (Å, °)

**Table:**

Br1—C1	1.906 (2)	C11—H11A	0.9900
O1—C19	1.210 (2)	C11—H11B	0.9900
O2—C18	1.214 (2)	C12—C17	1.388 (3)
O3—C26	1.209 (2)	C12—C13	1.394 (3)
N1—C9	1.445 (3)	C13—C14	1.389 (3)
N1—C27	1.466 (3)	C13—H13A	0.9500
N1—C8	1.473 (3)	C14—C15	1.390 (4)
C1—C2	1.379 (3)	C14—H14A	0.9500
C1—C6	1.383 (3)	C15—C16	1.380 (3)
C2—C3	1.390 (3)	C15—H15A	0.9500
C2—H2A	0.9500	C16—C17	1.396 (3)
C3—C4	1.395 (3)	C16—H16A	0.9500
C3—H3A	0.9500	C17—C18	1.472 (3)
C4—C5	1.399 (3)	C19—C20	1.479 (3)
C4—C7	1.510 (3)	C20—C21	1.392 (3)
C5—C6	1.390 (3)	C20—C25	1.398 (3)
C5—H5A	0.9500	C21—C22	1.380 (3)
C6—H6A	0.9500	C21—H21A	0.9500
C7—C8	1.546 (3)	C22—C23	1.403 (3)
C7—C10	1.553 (3)	C22—H22A	0.9500
C7—H7A	1.0000	C23—C24	1.387 (3)
C8—H8A	0.9900	C23—H23A	0.9500
C8—H8B	0.9900	C24—C25	1.390 (3)
C9—C19	1.545 (3)	C24—H24A	0.9500
C9—C26	1.553 (3)	C25—C26	1.488 (3)
C9—C10	1.583 (3)	C27—H27A	0.9800
C10—C18	1.544 (3)	C27—H27B	0.9800
C10—C11	1.546 (3)	C27—H27C	0.9800
C11—C12	1.512 (3)		
			
C9—N1—C27	116.69 (17)	C17—C12—C13	119.36 (19)
C9—N1—C8	107.46 (16)	C17—C12—C11	111.48 (16)
C27—N1—C8	114.35 (16)	C13—C12—C11	129.16 (19)
C2—C1—C6	121.85 (19)	C14—C13—C12	118.4 (2)
C2—C1—Br1	118.66 (16)	C14—C13—H13A	120.8
C6—C1—Br1	119.48 (15)	C12—C13—H13A	120.8
C1—C2—C3	118.85 (19)	C13—C14—C15	121.6 (2)
C1—C2—H2A	120.6	C13—C14—H14A	119.2
C3—C2—H2A	120.6	C15—C14—H14A	119.2
C2—C3—C4	121.30 (18)	C16—C15—C14	120.4 (2)
C2—C3—H3A	119.4	C16—C15—H15A	119.8
C4—C3—H3A	119.4	C14—C15—H15A	119.8
C3—C4—C5	118.01 (18)	C15—C16—C17	117.9 (2)
C3—C4—C7	122.69 (18)	C15—C16—H16A	121.1
C5—C4—C7	119.30 (18)	C17—C16—H16A	121.1
C6—C5—C4	121.49 (19)	C12—C17—C16	122.24 (19)
C6—C5—H5A	119.3	C12—C17—C18	109.19 (17)
C4—C5—H5A	119.3	C16—C17—C18	128.53 (19)
C1—C6—C5	118.46 (18)	O2—C18—C17	127.45 (19)
C1—C6—H6A	120.8	O2—C18—C10	125.27 (18)
C5—C6—H6A	120.8	C17—C18—C10	107.28 (16)
C4—C7—C8	115.99 (17)	O1—C19—C20	126.71 (18)
C4—C7—C10	114.55 (15)	O1—C19—C9	126.11 (19)
C8—C7—C10	104.92 (15)	C20—C19—C9	107.17 (16)
C4—C7—H7A	106.9	C21—C20—C25	120.93 (19)
C8—C7—H7A	106.9	C21—C20—C19	129.02 (18)
C10—C7—H7A	106.9	C25—C20—C19	110.05 (17)
N1—C8—C7	105.26 (15)	C22—C21—C20	118.20 (19)
N1—C8—H8A	110.7	C22—C21—H21A	120.9
C7—C8—H8A	110.7	C20—C21—H21A	120.9
N1—C8—H8B	110.7	C21—C22—C23	120.8 (2)
C7—C8—H8B	110.7	C21—C22—H22A	119.6
H8A—C8—H8B	108.8	C23—C22—H22A	119.6
N1—C9—C19	115.00 (16)	C24—C23—C22	121.3 (2)
N1—C9—C26	117.37 (16)	C24—C23—H23A	119.4
C19—C9—C26	101.32 (15)	C22—C23—H23A	119.4
N1—C9—C10	100.95 (15)	C23—C24—C25	117.79 (19)
C19—C9—C10	112.11 (15)	C23—C24—H24A	121.1
C26—C9—C10	110.47 (16)	C25—C24—H24A	121.1
C18—C10—C11	103.44 (15)	C24—C25—C20	120.99 (19)
C18—C10—C7	113.34 (16)	C24—C25—C26	129.75 (18)
C11—C10—C7	118.79 (16)	C20—C25—C26	109.26 (18)
C18—C10—C9	111.89 (15)	O3—C26—C25	126.98 (19)
C11—C10—C9	109.65 (15)	O3—C26—C9	125.77 (19)
C7—C10—C9	99.93 (14)	C25—C26—C9	107.22 (15)
C12—C11—C10	103.93 (16)	N1—C27—H27A	109.5
C12—C11—H11A	111.0	N1—C27—H27B	109.5
C10—C11—H11A	111.0	H27A—C27—H27B	109.5
C12—C11—H11B	111.0	N1—C27—H27C	109.5
C10—C11—H11B	111.0	H27A—C27—H27C	109.5
H11A—C11—H11B	109.0	H27B—C27—H27C	109.5
			
C6—C1—C2—C3	−2.2 (3)	C14—C15—C16—C17	−1.9 (3)
Br1—C1—C2—C3	177.23 (14)	C13—C12—C17—C16	0.6 (3)
C1—C2—C3—C4	0.5 (3)	C11—C12—C17—C16	−179.99 (19)
C2—C3—C4—C5	1.2 (3)	C13—C12—C17—C18	178.51 (18)
C2—C3—C4—C7	−179.53 (18)	C11—C12—C17—C18	−2.1 (2)
C3—C4—C5—C6	−1.2 (3)	C15—C16—C17—C12	1.2 (3)
C7—C4—C5—C6	179.50 (17)	C15—C16—C17—C18	−176.3 (2)
C2—C1—C6—C5	2.2 (3)	C12—C17—C18—O2	168.6 (2)
Br1—C1—C6—C5	−177.24 (14)	C16—C17—C18—O2	−13.7 (3)
C4—C5—C6—C1	−0.4 (3)	C12—C17—C18—C10	−11.8 (2)
C3—C4—C7—C8	40.8 (2)	C16—C17—C18—C10	165.9 (2)
C5—C4—C7—C8	−139.97 (18)	C11—C10—C18—O2	−160.17 (19)
C3—C4—C7—C10	−81.7 (2)	C7—C10—C18—O2	−30.2 (3)
C5—C4—C7—C10	97.5 (2)	C9—C10—C18—O2	81.9 (2)
C9—N1—C8—C7	−25.6 (2)	C11—C10—C18—C17	20.3 (2)
C27—N1—C8—C7	−156.81 (17)	C7—C10—C18—C17	150.23 (16)
C4—C7—C8—N1	−131.75 (17)	C9—C10—C18—C17	−97.69 (18)
C10—C7—C8—N1	−4.3 (2)	N1—C9—C19—O1	−29.7 (3)
C27—N1—C9—C19	−65.1 (2)	C26—C9—C19—O1	−157.37 (19)
C8—N1—C9—C19	164.97 (16)	C10—C9—C19—O1	84.8 (2)
C27—N1—C9—C26	53.9 (2)	N1—C9—C19—C20	149.06 (16)
C8—N1—C9—C26	−76.0 (2)	C26—C9—C19—C20	21.41 (19)
C27—N1—C9—C10	174.03 (16)	C10—C9—C19—C20	−96.38 (18)
C8—N1—C9—C10	44.09 (18)	O1—C19—C20—C21	−16.3 (3)
C4—C7—C10—C18	−83.5 (2)	C9—C19—C20—C21	164.93 (19)
C8—C7—C10—C18	148.17 (16)	O1—C19—C20—C25	163.7 (2)
C4—C7—C10—C11	38.2 (2)	C9—C19—C20—C25	−15.0 (2)
C8—C7—C10—C11	−90.1 (2)	C25—C20—C21—C22	−0.1 (3)
C4—C7—C10—C9	157.30 (16)	C19—C20—C21—C22	180.0 (2)
C8—C7—C10—C9	28.97 (18)	C20—C21—C22—C23	−1.0 (3)
N1—C9—C10—C18	−164.44 (15)	C21—C22—C23—C24	1.1 (3)
C19—C9—C10—C18	72.7 (2)	C22—C23—C24—C25	−0.1 (3)
C26—C9—C10—C18	−39.5 (2)	C23—C24—C25—C20	−1.0 (3)
N1—C9—C10—C11	81.38 (17)	C23—C24—C25—C26	178.8 (2)
C19—C9—C10—C11	−41.5 (2)	C21—C20—C25—C24	1.1 (3)
C26—C9—C10—C11	−153.72 (15)	C19—C20—C25—C24	−178.89 (18)
N1—C9—C10—C7	−44.19 (17)	C21—C20—C25—C26	−178.72 (18)
C19—C9—C10—C7	−167.09 (16)	C19—C20—C25—C26	1.3 (2)
C26—C9—C10—C7	80.71 (17)	C24—C25—C26—O3	14.7 (4)
C18—C10—C11—C12	−20.64 (19)	C20—C25—C26—O3	−165.4 (2)
C7—C10—C11—C12	−147.24 (17)	C24—C25—C26—C9	−166.9 (2)
C9—C10—C11—C12	98.86 (17)	C20—C25—C26—C9	13.0 (2)
C10—C11—C12—C17	14.9 (2)	N1—C9—C26—O3	31.6 (3)
C10—C11—C12—C13	−165.8 (2)	C19—C9—C26—O3	157.7 (2)
C17—C12—C13—C14	−1.7 (3)	C10—C9—C26—O3	−83.3 (2)
C11—C12—C13—C14	179.1 (2)	N1—C9—C26—C25	−146.80 (17)
C12—C13—C14—C15	0.9 (3)	C19—C9—C26—C25	−20.7 (2)
C13—C14—C15—C16	0.9 (3)	C10—C9—C26—C25	98.26 (18)

### Hydrogen-bond geometry (Å, °)

**Table:**

*D*—H···*A*	*D*—H	H···*A*	*D*···*A*	*D*—H···*A*
C11—H11B···O1	0.99	2.40	3.053 (2)	123
C15—H15A···O2^i^	0.95	2.44	3.163 (3)	133
C22—H22A···O3^i^	0.95	2.57	3.225 (3)	126

Symmetry codes: (i) *x*+1/2, −*y*−1/2, *z*+1/2.
